# Association of eNOS and Cav-1 gene polymorphisms with susceptibility risk of large artery atherosclerotic stroke

**DOI:** 10.1371/journal.pone.0174110

**Published:** 2017-03-27

**Authors:** Hann-Yeh Shyu, Ming-Hua Chen, Yi-Hsien Hsieh, Jia-Ching Shieh, Ling-Rong Yen, Hsiao-Wei Wang, Chun-Wen Cheng

**Affiliations:** 1 Section of Neurology, Department of Internal Medicine, Armed Forces Taoyuan General Hospital, Taoyuan, Taiwan; 2 Institute of Biology and Anatomy, National Defense Medical Center, Taipei, Taiwan; 3 Department of Neurology, Tri-Service General Hospital, National Defense Medical Center, Taipei, Taiwan; 4 Institute of Biochemistry, Microbiology and Immunology, Chung Shan Medical University, Taichung, Taiwan; 5 Department of Biomedical Sciences, Chung Shan Medical University, Taichung, Taiwan; 6 Clinical Laboratory, Chung Shan Medical University Hospital, Taichung, Taiwan; Universidad Francisco de Vitoria, SPAIN

## Abstract

Endothelial nitric oxide synthase (*eNOS*) is localized in caveole and has important effects on caveolar coordination through its interaction with caveolin-1 (*Cav-1*), which supports normal functioning of vascular endothelial cells. However, the relationship between genotypic polymorphisms of *e-NOS* and *Cav-1* genes and ischemic stroke (IS) remains lesser reported. This hospital-based case-control study aimed to determine the genetic polymorphisms of the *eNOS* (*Glu298Asp*) and *Cav-1* (*G14713A* and *T29107A*) genes in association with susceptibility risk in patients who had suffered from a large artery atherosclerotic (LAA) stroke. Genotyping determination for these variant alleles was performed using the TaqMan assay. The distributions of observed allelic and genotypic frequencies for the polymorphisms were in Hardy-Weinberg equilibrium in healthy controls. The risk for an LAA stroke in the *Asp298* variant was 1.72 (95% CI = 1.09–2.75) versus *Glu298* of the *eNOS*. In the *GA/AA* (rs3807987) variant, it was 1.79 (95% CI = 1.16–2.74) versus *GG* and in *TA/AA* (rs7804372) was 1.61 (95% CI = 1.06–2.43) versus *TT* of the *Cav-1*, respectively. A tendency toward an increased LAA stroke risk was significant in carriers with the *eNOS Glu298Asp* variant in conjunction with the *G14713 A* and *T29107A* polymorphisms of the *Cav-1* (aOR = 2.03, P-_trend_ = 0.002). A synergistic effect between *eNOS* and *Cav-1* polymorphisms on IS risk elevation was significantly influenced by alcohol drinking, heavy cigarette smoking (P-_trend_<0.01), and hypercholesterolemia (P-_trend_ < 0.001). In conclusion, genotypic polymorphisms of the *eNOS Glu298Asp* and *Cav-1 14713A/29107A* polymorphisms are associated with the elevated risk of LAA stroke among Han Chinese in Taiwan.

## Introduction

Despite the growing optimism due to recent advances in stroke therapy, strokes remain a major leading cause of disability and the third-leading cause of death among ethnic Chinese in Taiwan. An ischemic stroke (IS) is the most prevalent type of stroke, accounting for more than 80% of all stroke cases [1]. IS pathogenesis is believed to be multi-factorial, influenced by smoking, alcohol intake, hypertension, obesity, diabetes mellitus, and environmental factors as well as interactions with genetic components [2–4]. Recent progress in the understanding of the mechanism underlying the pathogenesis of cardiomyopathy has led to the discovery of therapeutic targets of nitric oxide (NO) with hopeful optimism [5]. Hence, gaining insight into the identification of genetic factors regarding NO has become an important issue to predict risk susceptibility and improve prevention strategies to reduce the human and economic burden of strokes.

Vascular endothelial NO synthesis is produced by the action of endothelial NO synthase (*eNOS*), whose encoded gene is located on chromosome 7q35. Endothelium-derived NO plays several predominant roles in arteries, including inhibition of the adhesion of platelets and leukocytes, vasodilation in the endothelium, and the reduction of the genesis of oxidized low-density lipoprotein to prevent atherogenesis [6–8]. In recent, eNOS has been reported to occur specifically in the subdomain within caveolin-1, encoded by the *Cav-1* gene, which assembles as a coat and scaffolding protein in caveolae and is responsible for the multiple phenotypes in vascular endothelial cells. Membrane caveolae consists of caveolins, a cholesterol-binding family with 21–24 kDa integral membrane proteins [9–11]. Binding affinity of eNOS/Cav-1 has multifunctional signaling in NO release which may account for the modified effect of Cav-1 in association with eNOS activity on vasodilation [12].

An individual vulnerability of *eNOS* and *Cav-1* polymorphisms to the pathogenesis among cardiovascular diseases has been studied [13–17], yet the correlation between genotypic polymorphisms of these two genes and the synergistic interaction of *eNOS*/*Cav-1* on susceptible risk of large artery atherosclerotic (LAA) stroke has never been reported. Thus, this hospital-based case-control study aimed to determine the comparative distribution of *eNOS* (*G894T*) and *Cav-1* (*G14713A* and *T29107A*) polymorphisms in patients who had undergone an LAA stroke and control subjects free of neurological diseases. In addition, the modified effects of the traditional risk factors, such as hypercholesterolemia, alcohol drinking, and heavy cigarette smoking on the genetic predisposition to have a stroke were evaluated.

## Materials and methods

### Study population

The sample size that was required in this hospital-based case-control study was computed by using “Genetic Power Calculator” (http://pngu.mgh.harvard.edu/) under the assumption of 80% statistical power, 5% variant allele frequency, 1:1 case/control ratio, and 5% error rate in an allelic test. The present study consisted of 229 consecutive patients who had undergone an LAA stroke and 243 age- and gender-matched healthy controls. IS was defined according to the World Health Organization definition as “rapidly developing clinical signs of focal (or global) disturbance of cerebral function lasting more than 24 hours with no apparent cause other than of vascular origin.” Any patients with atypical symptoms, including transient ischemic attacks, intracranial hemorrhage, post seizure palsy, brain trauma, brain tumors and younger stroke (<50 years old) were excluded from the study. The age at onset is defined as the age at which the first symptom of IS become evident by patient recollection. IS patients underwent at least one brain imaging (CT and/or MRI) study. Based on the clinical features and the information from brain imaging, echocardiography, ultrasonography of extracranial and intracranial arteries, and angiography (MRA or conventional angiography), these IS patients were sub-classified according to the TOAST classification system [18, 19]. The healthy control group was recruited from the same hospital in the health examination clinic during the same study period, and each of these controls was chosen on the basis that they have no evidence of any cerebrovascular disorders. Informed consent from each individual was obtained prior to specimen acquisition and the study design was approved by the Ethics Committee of Institutional Review Board of the Tri-Service General Hospital (TY101-11), Taipei, Taiwan.

### Data collection

The information collected included demographic characteristics (ethnic background, residence area, and educational level) and age at diagnosis. The definitions of hypertension and diabetes mellitus (DM) followed guidelines of Hypertension Management Guide for Doctors [20] and guided by Diagnosis and classification of diabetes mellitus of American Diabetes Association [21], respectively. Patients with hypertension or DM were assured by the presence of an actual diagnosis as well as received antihypertensive/antidiabetic medication. History of alcoholism (defined as alcohol abuse disorder according to the Diagnostic and Statistical Manual of Mental Disorders, 4th edition (DSM-IV) [22]), a current habit of daily cigarette smoking (heavy smokers consumed ≧10 cigarettes daily for at least 10 years), and the categorization of hypercholesterolemia as demonstrated in our previous report [23].

### DNA extraction and genotype analysis

A sample of 10-ml of peripheral blood, collected in acetate-citrate dextrose, was obtained from a fasting venous blood sample drawn from each patient and control subject. Genomic DNA was extracted from peripheral blood lymphocytes using a QIAamp DNA blood mini kit (QIAGEN Inc., Chatsworth, CA, USA) following the manufacturer’s protocol and stored at -20°C for subsequent genotype determination. These variant genotypes of interest were chosen for genotyping based on the following criteria: (i) the single nucleotide polymorphism (SNP) resulted in an amino acid substitution; (ii) the frequency of the variant allele was greater than five percent in the general population. Polymorphisms in the *Cav-1 G14713A* (rs3807987) and *T29107A* (rs7804372), and *e-NOS Glu298Asp* (rs1799983) genes were selected based on the information in the Chinese population arranged in the HapMap database (http://www.hapmap.org/). DNA sequences of probes and primers for the genotyping assays were chosen using the TaqMan Assays-by-Design^™^ supplied by Applied Biosystems (ABI, Foster City, CA, USA). Genotype analyses for these variant alleles were performed using the TaqMan assay and PCR amplification conditions on ABI Prism 7000 Sequence Detection System (Applied Biosystems, Foster City, CA). For quality assurance, independent positive and negative control samples were included and analyzed on each 96-well plate in each batch. To ensure that the observed polymorphisms were specific and not the result of experimental variations, all uncertain genotypic results were subjected to a repeating assay for confirmation.

### Statistical analyses

The means and standard deviations or frequency and percent were calculated for continuous and categorical variables, respectively. Univariate and multivariate analyses were used to determine socioeconomic statuses and risk factors for IS. Hardy-Weinberg equilibrium (HWE) was assessed using a chi-square test. The allelic and genotypic frequencies of each polymorphism for these four genes were compared between the cases and the control subjects using chi-square or Fisher’s exact test as appropriate. The adjusted odds ratios (aORs) and the corresponding 95% confidence intervals (95% CIs) for the association between polymorphisms in the *Cav-1* and *eNOS* genes and LAA IS risk were estimated considering age and gender in multivariate logistic regression analysis. All statistical tests were performed with SPSS version 19.0 based on a 2-tailed probability and *P*-values less than 0.05 were considered statistically significant.

## Results

Demographic characteristics of cases and control subjects are summarized in Table 1. There was no significant difference between the two groups in their age (stroke patients and controls were 73.2 ± 9.9 and 72.8 ± 7.9 years old, respectively) and gender. An increased LAA stroke risk was associated with risk factors, including hypertension (aOR = 3.17; 95% CI = 2.15–4.65), diabetes (aOR = 2.80; 95% CI = 1.83–4.28), hypercholesterolemia (aOR = 1.45; 95% CI = 1.01–2.08) using multiple logistic regression analysis after adjusting for age and sex. Besides, a significant risk elevation of a stroke was observed for patients with a cigarette smoking habit (aOR = 2.52; 95% CI = 1.62–3.94) (Table 1).

**Table 1:** Demographic data for the LAA stroke patients and control subjects.

	IS patients (n = 229)		Controls (n = 243)		OR (95% CI) ^a^	P-value
	n (%)	Mean±SD	n (%)	Mean±SD		
Age		73.2±9.9		72.8±7.9		
Gender					1.32 (0.90–1.92)	0.152
Men	154 (67.2)		148 (60.9)			
Women	75 (32.8)		95 (39.1)			
Hypertension					3.17 (2.15–4.65)	< 0.001
Yes	165 (72.1)		109 (44.9)			
Null	64 (27.9)		134 (55.1)			
Diabetes mellitus					2.80 (1.83–4.28)	< 0.001
Yes	86 (37.6)		43 (17.7)			
Null	143 (62.4)		200 (82.3)			
Hypercholesterolemia ^b^					1.45 (1.01–2.08)	0.047
Yes	118 (51.5)		103 (42.4)			
Null	111 (48.5)		140 (57.6)			
Gout					1.97 (1.04–3.76)	0.038
Yes	28 (12.2)		16 (6.6)			
Null	201 (87.8)		227 (93.4)			
Alcoholism ^b^					1.41 (0.77–2.58)	0.260
Yes	27 (11.8)		21 (8.6)			
Null	202 (88.2)		222 (91.4)			
Heavy cigarette smoking ^b^					2.52 (1.62–3.94)	< 0.001
Yes	73 (31.9)		38 (15.6)			
Null	156 (68.1)		205 (84.4)			

^a^ adjusted for age and sex.

^b^ disease status of hypertension, diabetes mellitus, and hypercholesterolemia, history of alcoholism and cigarette smoking were defined in the Materials and methods.

The allelic and genotypic frequencies of *e-NOS* (*G894T*) and *Cav-1* (*G14713A* and *T29107A*) polymorphisms are shown in Table 2. The frequencies of observed alleles and genotypes for these polymorphisms were in Hardy-Weinberg proportions in the controls. On the basis of inferred phenotypic manifestations [10, 24–26], subjects who carried rare alleles of the *eNOS 894T* (coded for Aspartate), *Cav-1 14713A*, and *29107A* were grouped together and considered a risk genotype in the following statistical analysis. For subjects who carried the homozygous *eNOS GG* genotype as a reference, a LAA stroke risk elevation was significant in carriers of combined *GT/TT* variant genotypes (OR = 1.72, 95% CI = 1.09–2.75). For those subjects who carried the homozygous *Cav-1 14713GG* genotype as a reference, we found aORs of 1.71 (95% CI = 1.08–2.69) for *GA*, 2.34 (95% CI = 0.90–5.92) for *AA*, and 1.79 (95% CI = 1.16–2.74) for the combined *GA*/*AA* genotypes in the multiple logistic regression model. Similar to the *Cav-1 T29107A* polymorphism, the combined *TA*/*AA* genotype was correlated, showing a significant association with an increased IS risk (aOR = 1.61; 95% CI = 1.06–2.43) compared to the *TT* genotype (Table 2).

**Table 2:** Genetic polymorphisms of *eNOS* and *Cav-1* genes in association with LAA stroke risk.

Gene and SNP ^a^	Cases (%)	CTLs (%)	cOR (95% CI)^b^	aOR (95% CI)^c^	aOR (95% CI)^d^
*e-NOS G894T* (rs1799983, Glu/Asp)					
*GG*	151 (65.9)	185 (76.1)	1.00 (Ref)^e^	1.00 (Ref)	1.00 (Ref.)
*GT*	62 (27.1)	51 (21.0)	1.49 (0.97–2.29)	1.52 (0.93–2.46)	1.72 (1.09–2.75)*
*TT*	16 (7.0)	7 (2.9)	2.80 (1.12–6.98)*	3.45 (1.25–9.55)*	
*G allele*	364 (79.5)	421 (86.6)	1.00 (Ref)		
*T allele*	94 (20.5)	65 (13.4)	1.67 (1.18–2.36)*		
*Cav-1 G14713A* (rs3807987)					
*GG*	138 (60.3)	174 (71.6)	1.00 (Ref)	1.00 (Ref)	1.00 (Ref.)
*GA*	75 (32.7)	60 (24.7)	1.57 (1.05–2.36)*	1.71 (1.08–2.69)*	1.79 (1.16–2.74)**
*AA*	16 (7.0)	9 (3.7)	2.24 (0.96–5.23)	2.34 (0.90–5.92)	
*G allele*	351 (76.6)	408 (84.0)	1.00 (Ref)		
*A allele*	107 (23.4)	78 (16.0)	1.59 (1.15–2.21)*		
*Cav-1 T29107A* (rs7804372)					
*TT*	128 (55.9)	167 (68.7)	1.00 (Ref)	1.00 (Ref)	1.00 (Ref.)
*TA*	82 (35.8)	65 (26.7)	1.65 (1.11–2.45)*	1.58 (1.01–2.45)*	1.61 (1.06–2.43)*
*AA*	19 (8.3)	11 (4.6)	2.25 (1.04–4.90)*	1.70 (0.71–4.06)	
*T allele*	338 (73.8)	399 (82.1)	1.00 (Ref)		
*A allele*	120 (26.2)	87 (17.9)	1.63 (1.19–2.22)*		

^a^ The ‘rs’ number shown is the National Center for Biotechnology Information dbSNP cluster ID for each SNP.

^b^ cOR, crude odd ratio;

^c^ aOR, statistical analysis was calculated by unconditional logistic regression and adjusted for age, gender, alcohol drinking, cigarette smoking, and disease status of diabetes mellitus, gout, hypertension, and hypercholesterolemia.

^d^ In these regression models, heterozygous and homozygous variants were grouped together and compared to the homozygous wild-type variant.

^e^ Ref, reference group.

* *P* < 0.05,

** *P* < 0.01.

We evaluated interactions between the variant alleles and atherosclerotic factors by determining the genotype frequency of the polymorphisms among the LAA stroke patients and the controls. As summarized in Table 3, a greater proportion of the *eNOS 894GT* and *TT* variants was seen in cases, showing a 3.23-fold risk increase in patients with hypercholesterolemia (95% CI = 1.70–6.12) and a 3.50-fold risk in patients with a long-term habit of heavy cigarette smoking (95% CI = 1.75–6.99). Besides, carriers with the *Cav-1 G14713A* polymorphism showed an elevated IS risk among those subjects within the same categorization of the clinical parameters, including hypercholesterolemia (aOR = 2.46, 95% CI = 1.42–4.27) and heavy cigarette smoking (aOR = 3.46, 95% CI = 1.75–6.84), Similarly, observations of the effects of hypercholesterolemia and heavy cigarette smoking on LAA stroke risk elevation were statistically significant in patients with the *Cav-1T29107A* variant genotype (*TA*/*AA*) (*P* < 0.05) (Table 3).

**Table 3:** Effect of clinical variables on individual genotypic polymorphisms in elevated risk of LAA stroke disease.

Genotype	Hypercholesterolemia			Alcoholism			Heavy cigarette smoking		
		Ca/Co	aOR (95% CI) ^a^		Ca/Co	aOR (95% CI)		Ca/Co	aOR (95% CI)
*e-NOS G894T*									
*GG*	Null	74/99	1.00 (Ref)^b^	Null	142/174	1.00 (Ref)	Null	109/160	1.00 (Ref)
*GG*	Yes	77/86	1.20 (0.78–1.84)	Yes	9/11	1.01 (0.41–2.49)	Yes	42/25	2.47 (1.42–4.28)**
*GT/TT*	Null	37/41	1.21 (0.71–2.06)	Null	60/48	1.53 (0.99–2.38)	Null	47/45	1.53 (0.95–2.47)
*GT/TT*	Yes	41/17	3.23 (1.70–6.12)***	Yes	18/10	2.21 (0.99–4.93)	Yes	31/13	3.50 (1.75–6.99)***
*Cav-1 G14713A*									
*GG*	Null	70/100	1.00 (Ref)	Null	120/161	1.00 (Ref)	Null	96/150	1.00 (Ref)
*GG*	Yes	68/74	1.31 (0.84–2.06)	Yes	18/13	1.85 (0.88–3.94)	Yes	42/24	2.73 (1.56–4.80)***
*GA/AA*	Null	41/40	1.47 (0.86–2.49)	Null	82/61	1.80 (1.20–2.71)**	Null	60/55	1.71 (1.09–2.66)*
*GA/AA*	Yes	50/29	2.46 (1.42–4.27)**	Yes	9/8	1.51 (0.57–4.03)	Yes	31/14	3.46 (1.75–6.84)***
*Cav-1 T29107A*									
*TT*	Null	65/97	1.00 (Ref)	Null	115/154	1.00 (Ref)	Null	90/141	1.00 (Ref)
*TT*	Yes	63/70	1.34 (0.85–2.13)	Yes	13/13	1.34 (0.60–3.00)	Yes	38/26	2.29 (1.30–4.03)**
*TA/AA*	Null	46/43	1.60 (0.95–2.69)	Null	87/68	1.71 (1.15–2.55)**	Null	66/64	1.62 (1.05–2.49)*
*TA/AA*	Yes	55/33	2.49 (1.46–4.24)**	Yes	14/8	2.34 (0.95–5.77)	Yes	35/12	4.57 (2.25–9.26)***

^a^ aORs and 95% CIs were calculated in a logistic regression model after adjusting for age and gender of IS patients.

^b^ Ref, represents the reference group, indicating *894GG* in *e-NOS* as well as *14713GG* and *29107TT* in *Cav-1*, respectively.

* *P* < 0.05,

** *P* < 0.01 and

*** *P* < 0.001.

The eNOS/Cav-1 combination has been suggested to play a key function in endothelial hyperpermeability, and our hypothetic mechanism underlying ischemic contracture is attributed to Cav-1 acting on vascular hyperpermeability through eNOS inhibition to reduce NO levels, leading to vasoconstriction and ischemic damages of the vascular system. Support for this hypothesis came from the observation that multiple interactions of genes on LAA stroke risk elevation were present in patients carrying a combination of the susceptible genotypes. By using a dummy variable coding scheme [27], an increased IS risk was shown in individuals who carried *eNOS Glu894Asp* variants together with the *Cav-1* allelic variants of *G14713A* and *T29107A* (aOR = 4.20, 95% CI = 1.66–10.63). A significant trend toward LAA stroke risk elevation was found in patients carrying one additional risk genotype measured by the β estimation (*P*_*trend*_ = 0.002) (Table 4).

**Table 4:** Gene-to-gene interactions of *eNOS* and *Cav-1* polymorphisms in association with LAA stroke.

Genotypes		Cases (%)	Controls (%)	aOR (95% CI) ^a^
*eNOS G894T*	*Cav-1 G14713A*			
*GG*	*GG*	95 (41.5)	129 (53.1)	1.00 (Ref) ^b^
*GG*	*GA/AA*	56 (24.4)	56 (23.1)	1.47 (0.96–2.25)
*GT/TT*	*GG*	43 (18.8)	45 (18.5)	
*GT/TT*	*GA/AA*	35 (15.3)	13 (5.3)	4.12 (1.88–9.03)***
One additional risk-genotype			1.77 (1.28–2.44); *P*_interaction_ < 0.001	
*eNOS G894T*	*Cav-1 T29107A*			
*GG*	*TT*	91 (39.7)	126 (51.8)	1.00 (Ref)
*GG*	*TA/AA*	60 (26.2)	59 (24.3)	1.42 (0.93–2.19)
*GT/TT*	*TT*	37 (16.2)	41 (16.9)	
*GT/TT*	*TA/AA*	41 (17.9)	17 (7.0)	3.08 (1.52–6.25)**
One additional risk-genotype			1.63 (1.19–2.22) *P*_interaction_ = 0.002	
*eNOS 894GG*				
*Cav-1 G14713A*	*Cav-1 T29107A*			
*GG*	*TT*	78 (51.6)	110 (59.5)	1.00 (Ref)
*GG*	*TA/AA*	30 (19.9)	35 (18.9)	1.20 (0.69–2.13)
*GA/AA*	*TT*			
*GA/AA*	*TA/AA*	43 (28.5)	40 (21.6)	1.53 (0.91–2.58)
One additional risk-genotype			1.23 (0.96–1.59) *P*_-trend_ = 0.108	
*eNOS 894GT/TT*				
*Cav-1 G14713A*	*Cav-1 T29107A*			
*GG*	*TT*	29 (37.2)	36 (62.1)	1.00 (Ref)
*GG*	*TA/AA*	22 (28.2)	14 (24.1)	1.95 (0.85–4.46)
*GA/AA*	*TT*			
*GA/AA*	*TA/AA*	27 (34.6)	8 (13.8)	4.20 (1.66–10.63)**
One additional risk-genotype			2.03 (1.30–3.18) *P*_-trend_ = 0.002	

^a^ aORs and 95% CIs were calculated in multivariate logistic regression model considering factors of age of onset and sex.

^b^ Ref, reference group.

**, *P* < 0.01 and

***, *P* < 0.001

Based on the hypothetic mechanism that these high-risk genotypes were associated with an increasing risk of stroke disease by mimicking ischemic contracture on reduced vascular hyperpermeability to inhibit NO generation, the modified effects of hypercholesterolemia, alcohol drinking, and heavy cigarette smoking on LAA stroke risk would be more significant in carriers of high-risk genotypes of *eNOS* and *Cav-1*. We thus examined the possible interaction between the *eNOS Glu894Asp* and *Cav-1 G14713A* and *T29107A* polymorphisms in conjunction with the clinical features using the joint and stratified methods. As a result, among *eNOS Glu894Asp* carriers, individuals having variant alleles of the *Cav-1 G14713A* and *T29107A* variants were associated with an increasing LAA stroke risk when compared to those who carried the homozygous genotype of *14713GG* and *29107TT*. Moreover, the combined effect of the interaction between *eNOS* and *Cav-1* polymorphisms on the LAA stroke risk elevation was shown in those diagnosed with hypercholesterolemia (aOR = 12.26, 95% CI = 2.39–62.95), alcoholism (aOR = 4.97, 95% CI = 0.96–25.7), and heavy cigarette smoking (aOR = 8.61, 95% CI = 1.74–42.50); the P-value for the trend test in *eNOS 894Asp* carriers with one additional *Cav-1* polymorphism was significant, as measured by the β estimates from the regression model (Table 5).

**Table 5:** Risk elevation of an LAA stroke in individuals with the *eNOS 894Asp* variant genotype in combination with *Cav-1* polymorphisms stratified by hypercholesterolemia, alcoholism, or heavy cigarette smoking.

No. of *Cav-1* risk genotypes		Hypercholesterolemia			Alcoholism			Heavy cigarette smoking	
		aOR (95% CI) ^a^	*P*-value		aOR (95% CI)	*P*-value		aOR (95% CI)	*P*-value
0	Null	1.00 (Ref) ^b^		Null	1.00 (Ref)		Null	1.00 (Ref)	
0	Yes	2.94 (0.96–8.97)	0.057	Yes	1.05 (0.28–3.88)	0.960	Yes	1.32 (0.39–4.48)	0.658
1	Null	1.56 (0.49–4.93)	0.447	Null	1.77 (0.71–4.41)	0.222	Null	1.45 (0.52–4.48)	0.475
1	Yes	5.36 (1.57–18.28)	0.007	Yes	3.10 (0.55–17.55)	0.201	Yes	3.63 (1.02–13.03)	0.048
2	Null	4.30 (1.34–13.78)	0.014	Null	3.97 (1.37–11.51)	0.011	Null	3.07 (1.01–9.33)	0.049
2	Yes	12.26 (2.39–62.95)	0.003	Yes	4.97 (0.96–25.7)	0.056	Yes	8.61 (1.74–42.50)	0.008
Additive effect on IS risk elevation		1.51 (1.21–1.87)			1.41 (1.14–1.75)	0.002		1.43 (1.16–1.77)	0.001

^a^ aORs and 95% CIs were estimated in multivariate logistic regression model after adjusting for the age of onset and sex.

^b^ Ref, reference group; the risks were estimated using the subjects harboring no high-risk genotypes (*Cav-1 14713GG* and *29107TT* genotype) as the reference.

## Discussion

In this hospital-based case-control study, we carried out a comparative analysis of the frequency distribution of the *894T* allele in the *eNOS* gene in our control subjects with the reported frequency and confirmed a rare prevalence of the variant *T* allele, similar to the documented frequencies among Asian populations, including ethnic Taiwanese [28–30]. Our observation that a positive association between the *eNOS Glu298Asp* variant and LAA stroke is consistent with the findings that the *eNOS Glu298Asp* polymorphism is in association with coronary artery disease and myocardial infarction among different ethnic cohorts, including Korean, Italian, North Indian, Turkish, and Han-Chinese population [31–35]. These results provide justification of the mutated *894T* allele playing an independent risk factor for carotid atherosclerosis [25]. Further, because NO molecule derived from eNOS conversion in the vascular endothelium has the vasoprotective effects through elimination of superoxide radicals, reduction of platelet aggregation, anti-inflammation, and limiting the oxidation of atherogenic low-density lipoproteins [36]; thus, dysfunction of the endothelial NOS activity has an impact of oxidative stress on vascular endothelium in healthy smokers [37]. Therefore, our findings that individuals carried *eNOS Glu298Asp* genotype with hypercholesterolemia and with habits of drinking alcohol and heavy smoking cigarettes are interpreted by the presence of an individual impairment in the vasoprotective capability causally linked to the eNOS polymorphism.

The caveolar coordination of eNOS, more specifically its interaction with Cav-1, plays a key role in normal eNOS activity to generate vascular NO, which is a bioavailable controller of shear-dependent NO release [38]. Knockdown experiments of *Cav-1* have been shown to cause cardiohypertrophy, diabetes, and pulmonary and focal cerebral ischemia and reperfusion injury [39–41]. Recent genome wide association reports have shown for that *Cav-1* SNPs are associated with stroke disease and that the *Cav-1 G14713A* elevates risk of coronary artery disease and myocardial infarction [24, 42–44]. However, some results are conflicting regarding the association between the *Cav-1* polymorphisms and the risk of atrial fibrillation in cardiac arrhythmias [45]. In this study, we screened two SNPs, the *G14713A* and *T29107A* polymorphisms of the *Cav-1* gene and examined their genotypic association with LAA stroke susceptibility (Table 2). Remarkably, based on a combinatorial study with genetic-environmental consideration, our findings provide realistic data regarding the risk susceptibility of an LAA stroke modified by gene-to-gene interactions; the two-locus risk genotype *14713A*/*T29107A* at the *Cav-1* showed the highest odds ratio in combination with the *eNOS Glu298Asp* genotype (Table 4). It is likely that polymorphisms in the *Cav-1* gene increase high plasma triglyceride levels, resulting in endothelial function abnormality and ischemia [46]. It was reported that caveolae and caveolin-1 reverse cholesterol transport in atherosclerosis [47], and that the integration of eNOS and caveolin-1 principally participates in cholesterol trafficking, lipid homeostasis, and transmembrane signaling. An observation *in vitro* also indicated that the eNOS Glu298Asp variant alters caveolar localization in the microdomain of endothelial cells and function deficit in this eNOS corresponding enzyme casually linking Cav-1 highlights the relevance of interaction between eNOS and Cav-1 in reducing vasoprotection in arterial disease [43]. Therefore, molecular evidence of the interaction between *eNOS* and *Cav-1* polymorphisms, such as *14713A*, *T29107A* and *12759A* alleles, on risk predisposition in LAA strokes in carriers who have hypercholesterolemia should be required to provide support in justifying our current association reports.

Notably, future molecular studies are required to reveal the important roles of *eNOS Glu298Asp* and *Cav-1 14713A*/*29107A* regarding the development of the coronary atrial disease. In the genomic era of personalized medicine, population-based genotyping association studies that are precise can be done if individual data with consideration of the traditional vascular risk factors [48], including hypertension, atrial fibrillation, obesity, diabetes, hypercholesterolemia, hyperuricemia, alcoholism, and heavy cigarette smoking, are available. In conclusion, individuals’ susceptibility to an LAA stroke is influenced by genetic variants in caveolar genes in association with stroke-related risk factors. This study demonstrated that the *eNOS Glu298Asp* in combination with *14713A*/*29107A* at the *Cav-1* locus was associated with a significant risk of an LAA stroke.

## Supporting information

S1 Table Demographical characteristics (age, gender, presence of hypertension, history of alcoholism and cigarette smoking) and polymorphism data of stroke patients and control subjects. (XLS)

## Abbreviations

95% CI: 95% confidence interval
Cav-1: Caveolin-1
eNOS: Endothelial nitric oxide synthase
HWE: Hardy-Weinberg equilibrium
LAA stroke: Large artery atherosclerotic stroke
NO: Nitric oxide
OR: Odds ratio
SNP: Single nucleotide polymorphism
